# Epidermodysplasia Verruciformis: A Rare Case Report

**DOI:** 10.7759/cureus.9046

**Published:** 2020-07-07

**Authors:** Rasha Alshammari, Ahmed Al-Issa, Yasser A Ghobara

**Affiliations:** 1 Medicine and Surgery, University of Hail, College of Medicine, Hail, SAU; 2 Dermatology, Derma Clinic, Riyadh, SAU

**Keywords:** autosomal recessive, human papillomavirus (hpv), epidermodysplasia verruciformis

## Abstract

Epidermodysplasia verruciformis (EV) is an uncommon disorder that is transmitted in an autosomal recessive manner. It is characterized by increased susceptibility to human papillomavirus (HPV) infection, which presents with hypo- or hyperpigmented macular lesions, pityriasis versicolor-like lesions, and an early tendency to transform into skin cancer.

We present a case of a 36-year-old female with complaints of asymptomatic, multiform lesions over the face, neck, chest, and upper arms. Histopathology was suggestive of EV, and our patient was given oral isotretinoin 20 mg/day and advised strict photoprotection.

## Introduction

Epidermodysplasia verruciformis (EV) is an uncommon, lifetime, autosomal recessive genodermatosis disorder that affects the immune system. It is characterized by increased sensitivity to human papillomavirus (HPV) infection [1]. It occurs in two forms, sporadic or familial, and does not have a tendency toward a specific race or gender [2].

It is characterized by pityriasis versicolor-like macules, flat wart-like lesions, macules, and seborrheic keratosis-like plaques that begin in childhood mainly on the sun-exposed areas of the face, neck, trunk, and extremities [1,3]. There is an increased risk of the malignant transformation of these lesions especially on the sun-exposed areas [1].

We report a new EV case, and provide its brief documentation.

## Case presentation

**Figure 1:**
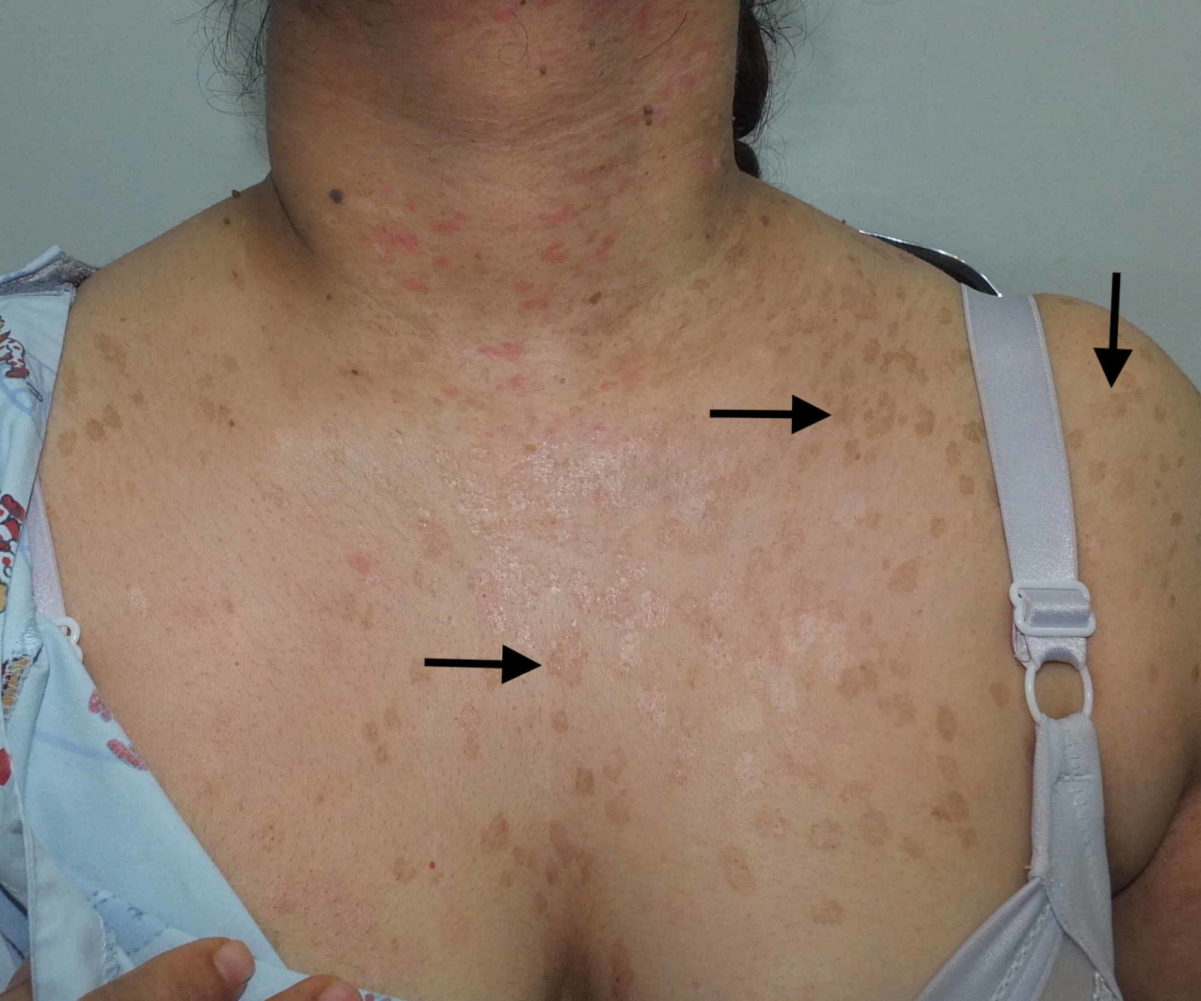
Multiple brownish, slightly scaly annular macules Arrows point to lesions of interest.

**Figure 2:**
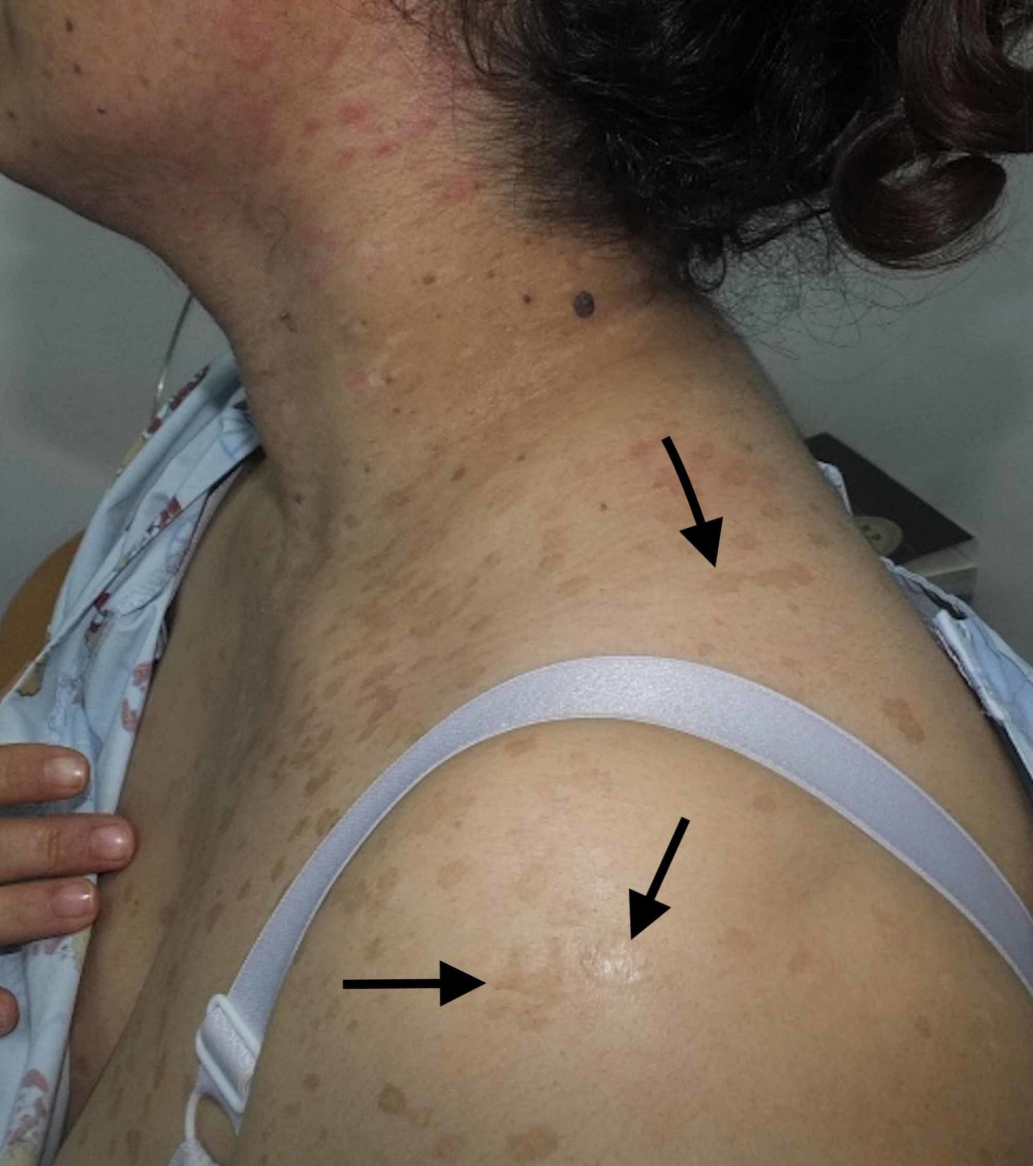
Multiple brownish, slightly scaly annular macules Arrows point to lesions of interest.

**Figure 3:**
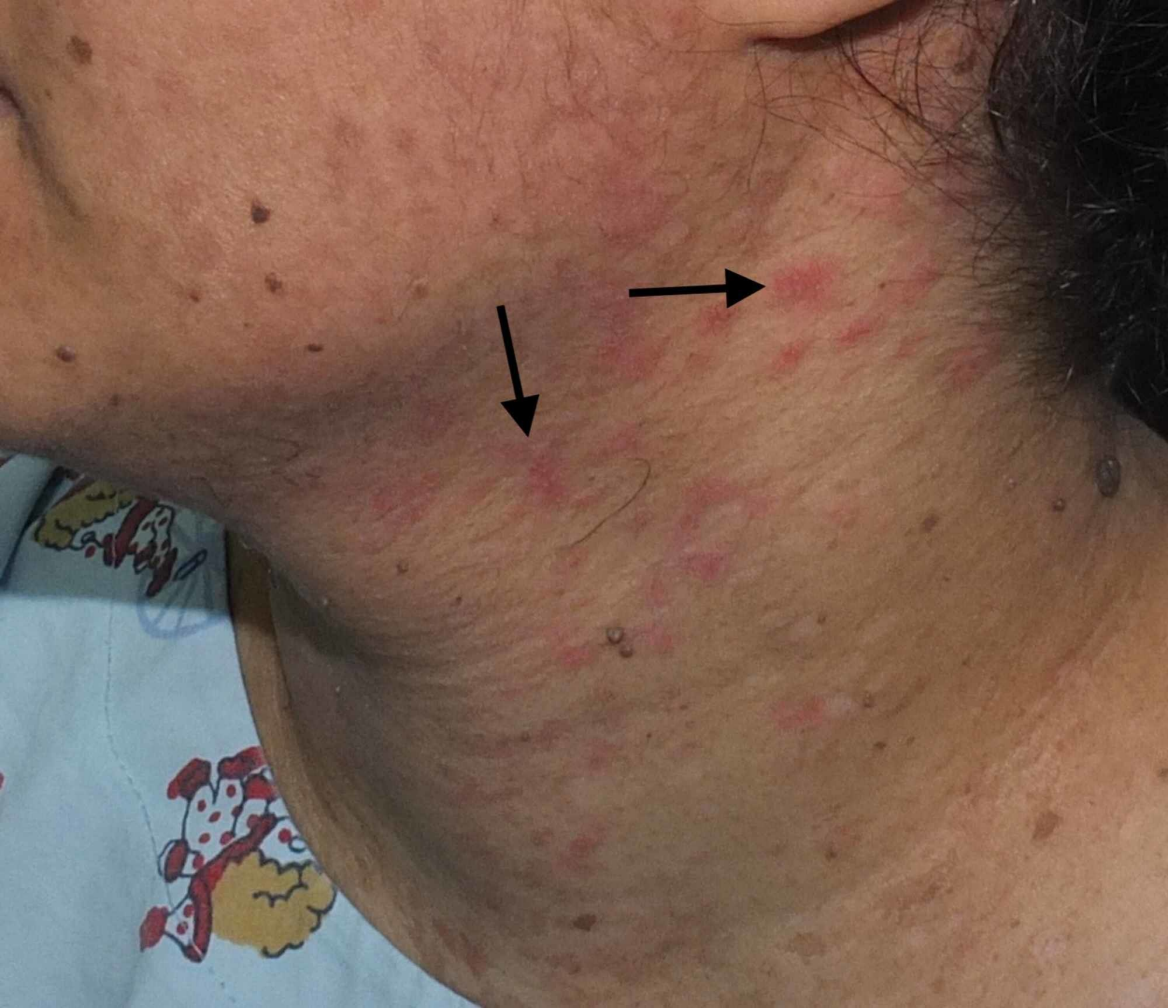
Multiple erythematous, slightly scaly papules on the neck Arrows point to lesions of interest.

**Figure 4:**
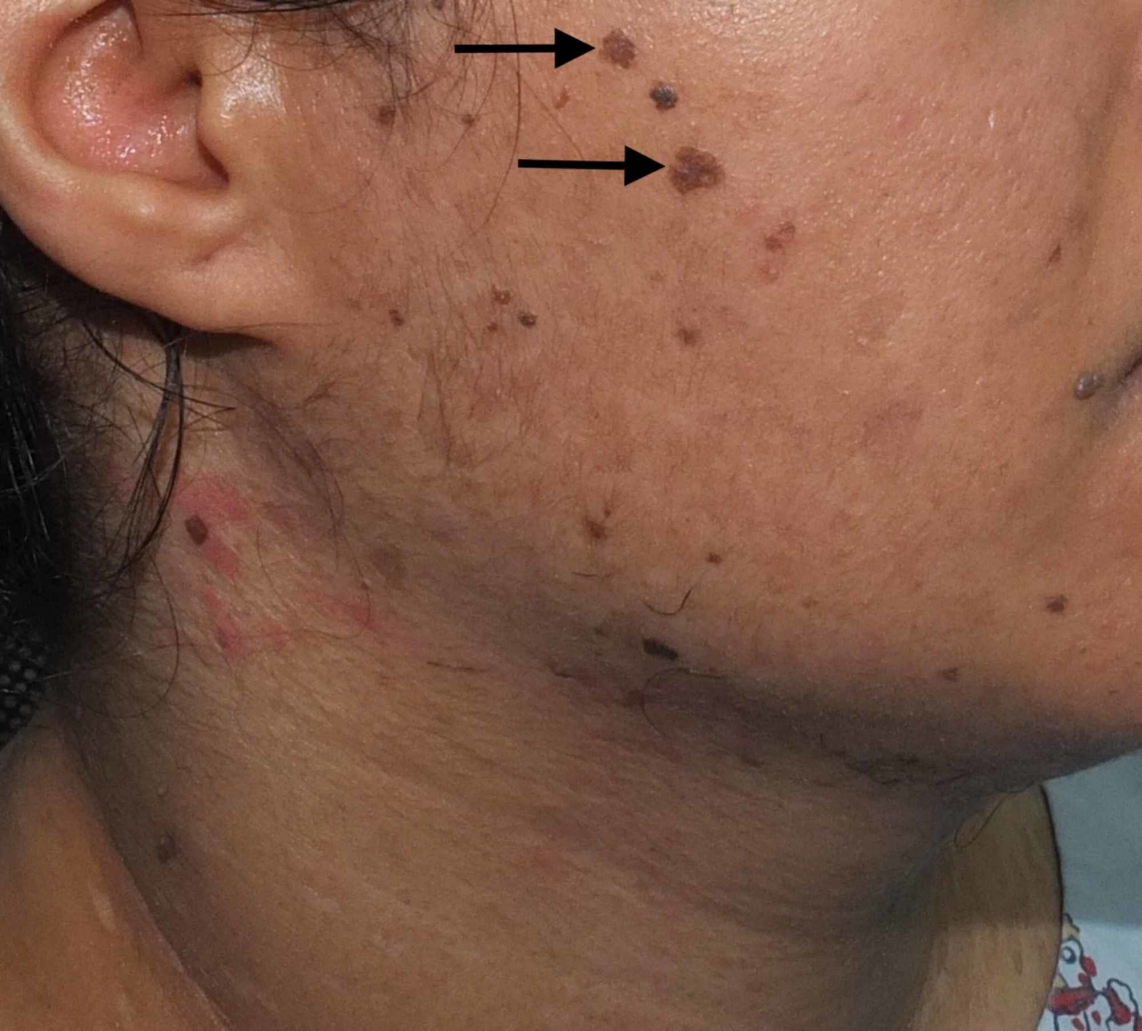
Seborrheic keratosis-like lesions on the face Arrows point to lesions of interest.

A 36-year-old female born of a non-consanguineous marriage presented to the dermatology clinic with complaints of asymptomatic lesions over the face, neck, chest, and upper arm since the age of 16 years. The lesions started on the chest, gradually increased in number, and finally spread to the neck, face, and upper arm. The patient had never consulted a doctor before and, hence, had not had any treatment for the skin lesions. There were no similar complaints in the family. She was otherwise healthy, with no past history of surgeries. On physical examination, there were multiple brownish, slightly scaly annular macules symmetrically distributed over the neck, chest, and upper arms (Figures 1-2), multiple erythematous, slightly scaly papules on the neck (Figure 3), and seborrheic keratosis-like lesions on the face (Figure 4). The hair, nails, mucous membrane, and other systemic examinations showed no abnormality. A punch skin biopsy was taken from the brownish slightly scaly annular macules over the left outer upper arm, which showed epidermal thickening with swollen cells in the upper epidermis. There are nests of large cells with a prominent perinuclear halo and grayish-blue cytoplasm. The stratum corneum shows a basket weave-like pattern, and some epidermal cells show dysplastic changes. The clinical presentation was characteristic, and the histopathological features were diagnostic of epidermodysplasia verruciformis. Baseline fasting lipid profile and liver function tests were done before starting oral retinoid, and the results were normal. The patient was advised strict photoprotection and started on oral isotretinoin 0.5 mg/kg. She has not completed her family and was advised to use two contraceptive methods. She was also advised regular follow-up.

## Discussion

Epidermodysplasia verruciformis is a lifetime disorder of cell-mediated immunity (CMI), with no preference for a specific sex, race, or geographic area [4]. It is an uncommon, autosomal recessive genodermatosis characterized by an increased risk of skin malignancy [5]. As mentioned earlier, the disease can be sporadic or familial. The familial form of EV is more common, but, in our case, there was no family history of the disease and, hence, we assume that she had a sporadic appearance. However, the autosomal dominant pattern of inheritance had been reported in two cases [3]. Epidermodysplasia verruciformis can be acquired and is usually seen in immunodeficient patients such as after renal transplantation, in Hodgkin’s disease and systemic lupus erythematosus, and with human immunodeficiency virus (HIV) infection [5]. 

In EV, there are mutations in the EVER1 or EVER2 genes located on chromosome 17q25, which, as a result of a defect of cell-mediated immunity, lead to unusual increased sensitivity of the patients to a distinct group of HPV genotypes recognized as EV HPV [1]. Intracellular zinc homeostasis is controlled by a complex of EVER proteins, and zinc transporter proteins have a role in suppressing EV expression [6]. The characteristic feature of EV is a specific defect of cell-mediated immunity that is demonstrated by the inhibition of natural cytotoxicity and the proliferation of T lymphocytes against HPV-infected squamous cells in EV skin lesions [7]. Ultraviolet (UV) B and X-rays are also important carcinogenic cofactors [6]. Frequent sun exposure, in addition to immunologic defects in patients with EV, is likely to induce mutations of the tumor suppressor gene protein (p53), which leads to the development of skin malignancy in adult patients [8]. Transformation into malignancy occurs in 35% to 50% of patients around 40 to 50 years of age, more commonly with Bowen’s-type carcinoma in situ followed by invasive squamous cell carcinoma; metastasis is uncommon [1]. Transformation into skin cancer depends mainly on the oncogenic potential of the infecting virus [3]. There are more than 30 EV-associated HPV types that have been recognized, including HPV5, HPV8, HPV12, HPV14, HPV15, HPV17, HPV19-HPV25, HPV36, HPV38, HPV47, and HPV50 [4]. Malignant transformations are primarily caused by HPV5 and HPV8 [9]. A young woman presented with a left infraorbital lesion that revealed squamous cell carcinoma [1].

Primary skin lesions are multiform, usually warty, lichenoid, and flat-topped papules [8]. At first, the lesions are confined, hypochromic scaly patches on the face and neck, resembling tinea versicolor [6]. Over a period of time, they increase in number and tend to develop into papules that look like flat warts, pink to brownish in color, a few millimeters in size, with a smooth surface, and later spread to the dorsum of the hands, forearms, knees, legs, and feet [6]. With disease progression, some lesions fade and new lesions may emerge on other parts of the body [6]. Findings are confined to the skin and uncommonly appear on the mucous membrane [8]. A case presented with varied forms of lesions, including multiple, slightly scaling, hypopigmented plaques, flat normochromic plaques, erythematous plaques with sharp edges, and actinic keratosis lesions on the face [2].

Presently, there is no specific and effective therapy for EV. Our aims for the management of EV are to prevent the progression of benign lesions into malignancy through preventive measures such as genetic counseling, photoprotection, and the monitoring of symptoms for the early detection of premalignant and malignant lesions. Patients should be advised to use sunblock from childhood [2]. In another case, zinc therapy was used for treating EV, with a response rate of 20% to 40%, without a recurrence after six months [10].

Pharmacologic treatments include oral and topical retinoids, interferon, immunotherapy, imiquimod, and cimetidine [1]. Presently, acitretin 0.5-1 mg/day is the drug of choice [3]. Oral retinoids (0.5 mg/kg) used in two sisters with an autosomal recessive pattern showed that cutaneous lesions decreased in size after a short time but a relapse of the lesions occurred after one year [9]. A surgical approach, including electrosurgical removal and cryotherapy, is also used to manage benign and premalignant lesions [6]. Malignant lesions were also surgically treated [6]. An EV patient had squamous cell carcinoma on the face treated surgically, and it was more effective [1]. As discussed, different treatment modalities are offered against EV; however, the most important are the education of the patient, early diagnosis, and excision of the premalignant and malignant lesions. These patients also need regular, life-long follow-up due to the high risk of developing premalignant and malignant lesions.

## Conclusions

The patterns of inheritance of epidermodysplasia verruciformis is autosomal recessive in most EV patients, although some patients can exhibit sporadic appearance. In the case described herein, the patient had parents that were not in a consanguineous marriage and were without EV lesions. In addition, she did not have family members with the same lesions and displayed a sporadic appearance. Extended exposure to sunlight and not using sunblock can precipitate the malignant transformation of EV lesions. Our patient did not use sunblock, and we recommended that she did so. Regular follow-up is also mandatory for her.
